# An increase in tumor-infiltrating lymphocytes after treatment is significantly associated with a poor response to neoadjuvant endocrine therapy for estrogen receptor-positive/HER2-negative breast cancers

**DOI:** 10.1007/s12282-023-01462-5

**Published:** 2023-04-28

**Authors:** Reiko Fukui, Takahiro Watanabe, Koji Morimoto, Yukie Fujimoto, Masayuki Nagahashi, Eri Ishikawa, Seiichi Hirota, Yasuo Miyoshi

**Affiliations:** 1 Department of Surgery, Division of Breast and Endocrine Surgery, School of Medicine, Hyogo Medical University, Mukogawa-Cho 1-1, Nishinomiya City, Hyogo 663-8501 Japan; 2 Department of Clinical Pathology, Chibune General Hospital, Osaka, Japan; 3 Department of Nutrition, College of Nutrition, Koshien University, Takarazuka, Hyogo Japan; 4 Department of Surgical Pathology, School of Medicine, Hyogo Medical University, Nishinomiya, Hyogo Japan

**Keywords:** Breast cancer, Endocrine therapy, Tumor-infiltrating lymphocytes, FOXP3, CD8

## Abstract

**Background:**

The reason for the poor prognosis of estrogen receptor (ER) + /human epidermal growth factor receptor 2 (HER2)− breast cancer patients with high levels of tumor-infiltrating lymphocytes (TILs) is poorly understood. The association between TILs and response to neoadjuvant endocrine therapy (NET) was examined.

**Methods:**

We recruited 170 patients with ER + /HER2− breast cancer who were treated with preoperative endocrine monotherapy. TILs were evaluated before and after NET, and their changes were noted. Furthermore, T cell subtypes were examined using CD8 and FOXP3 immunohistochemical analyses. Neutrophil and lymphocyte counts in the peripheral blood were analyzed with reference to TIL levels or changes. Responders were defined as Ki67 expression levels ≤ 2.7% after treatment.

**Results:**

Post-treatment (*p* = 0.016), but not pre-treatment (*p* = 0.464), TIL levels were significantly associated with the response to NET. TIL levels increased significantly after treatment among non-responders (*p* = 0.001). FOXP3 + T cell counts increased significantly after treatment in patients with increased TILs (*p* = 0.035), but not in those without increased TILs (*p* = 0.281). Neutrophil counts decreased significantly after treatment in patients without increased TILs (*p* = 0.026), but not in patients with increased TILs (*p* = 0.312).

**Conclusion:**

An increase in TILs after NET was significantly associated with a poor response to NET. Given that FOXP3 + T-cell counts increased, and neutrophil counts did not decrease in patients with increased TILs after NET, the induction of an immunosuppressive microenvironment was speculated to play a role in the inferior efficacy. These data might partially indicate the involvement of the immune response in the efficacy of endocrine therapy.

**Supplementary Information:**

The online version contains supplementary material available at 10.1007/s12282-023-01462-5.

## Introduction

Endocrine therapy (ET) is essential for the treatment of estrogen receptor (ER)-positive and human epidermal growth factor receptor 2 (HER2)-negative invasive breast cancer. In clinical practice, neoadjuvant endocrine therapy (NET) is an attractive treatment option for improving breast-conserving surgery because of the reduced tumor size resulting from ET [1]. In this context, clinical responses evaluated using the Response Evaluation Criteria in Solid Tumors (RECIST) are applicable to NET as treatment indicator [2]. However, since ET effectiveness is obtained not only by clinical response but also by improving prognosis in patients with ER+/HER2− breast cancer, other biomarkers for ET are needed. In a previous study on NET, low expression levels of the proliferative marker Ki67 after 2 weeks of treatment were significantly associated with longer recurrence-free survival (*p* = 0.008), but Ki67 levels at baseline were not (*p* = 0.07) [3]. Because ET suppresses cell cycle progression and induces Gap 1 phase arrest, Ki67 expression in cancer cells is speculated to be a more precise biomarker for ET than the RECIST evaluation. In line with this, Ianza et al. reported no significant correlation between clinical response and disease-free survival (DFS) (*p* = 0.84) or overall survival (OS) (*p* = 0.74) with letrozole-based neoadjuvant therapy, but the prognosis was significantly associated with the delta-Ki67 proliferation index (*p* = 0.002 for DFS; *p* = 0.009 for OS) [4]. Consequently, a decrease in Ki67 is accepted as a predictor of ET in clinical studies where cell cycle complete arrest, evaluated by Ki67 ≤ 2.7% after treatment, has been used as a biomarker for neoadjuvant endocrine-based therapy [5, 6]. Therefore, in clinical practice, the response to ET may be evaluated by the proliferative response determined by the downregulation of Ki67 expression levels.

Local immune markers such as tumor-infiltrating lymphocytes (TILs) have been established as prognostic and predictive factors for chemotherapy in triple-negative (TN) and HER2+ breast cancers [7]. In a meta-analysis that included 15,676 patients from 22 eligible clinical trials, a 10% increase in TILs was significantly associated with improved OS in HER2+ (hazard ratio [HR], 0.92; 95% confidence interval [CI] 0.89–0.95) and TN (HR 0.90; 95% CI 0.89–0.92) subtypes, but not in the ER+/HER2− subtype (HR 1.06; 95% CI 0.99–1.13) [8]. Contrary to TN and HER2 + subtypes, a significantly shorter survival period in patients with high TILs than in those with low TILs was observed in ER+/HER2− breast cancer (*p* = 0.026) [9]. Furthermore, Denkert et al. reported that among patients treated with NAC, higher levels of TILs were associated with shorter OS in the ER+/HER2- subtype (HR 1.10; 95% CI 1.02−1.19; *p* = 0.011) [10]. Since the positive association between pCR and TILs was consistently recognized in the ER + /HER2- subtype (pCR 6% for low TILs, 11% for intermediate TILs, and 28% for high TILs), the inferior prognosis of the ER+/HER2− subtype might not be due to the low efficacy of chemotherapy.

However, the relationship between TILs and their responses to ET has rarely been investigated. In a study by Lundgren et al., 2 year adjuvant tamoxifen (TAM) was compared with no TAM in 564 premenopausal patients according to TIL levels in archival tissues [11]. The breast cancer-free interval was significantly longer in the TAM group than in the control for patients with TILs < 50% (HR 0.63 95% CI 0.47−0.84; *p* = 0.002), but this association was absent in the TILs ≥ 50% group (HR 0.84; 95% CI 0.24−2.86; *p* = 0.77). Similarly, distant recurrence-free interval (DRFI) of patients treated with TAM was significantly improved compared with those with no TAM in ER + /HER2− postmenopausal breast cancer when TIL-low group (< 10%) was considered (HR 0.49, 95% CI 0.31–0.78, *p* = 0.002). But no significant benefit was obtained from TAM among patients with TIL-high group (≥ 10%) [12]. These data support the hypothesis that the inferior efficacy of ET in the TIL-high group of the ER + /HER2− subtype results in a poor prognosis. According to a study on neoadjuvant letrozole ± lapatinib, there was no statistically significant association between Ki67 suppression and high- and low-stromal TILs (− 81% vs − 66%; *p* = 0.513) [13]. Skriver et al. reported that among breast cancers with no pathological response, TILs were significantly increased after neoadjuvant letrozole (odds ratio [OR] 0.71; 95% CI 0.53−0.96;* p* = 0.02) [14]. In contrast, TILs were significantly increased in responders (mean%, 5.07 ± 10.42 vs. 3.047 ± 6.859; *p* = 0.0071) but not in non-responders (mean%, 3.15 ± 3.648 vs. 2.425 ± 4.919; *p* = 0.0938) [15]. Thus, the significance of TILs, including issues in baseline measurement and changes in sensitivity to ET, remains to be elucidated.

To determine the influence of the immune response on the efficacy of ET in ER + /HER2− breast cancer, we investigated TIL levels in samples obtained before and after the start of treatment in response to NET. In addition, T cell subsets were identified using markers of cluster of differentiation (CD)8 and forkhead box protein 3 (FOXP3) for cytotoxic (anti-tumor effects) T cells and regulatory (negatively regulated immune responses) T cells (Tregs), respectively, by immunohistochemical staining. To clarify the mechanisms underlying the association between TIL levels and response to NET, changes in TIL levels were compared with peripheral immune-related blood markers, including neutrophil and lymphocyte counts.

## Patients and methods

### Patient eligibility and NET

A total of 186 patients with histologically diagnosed invasive breast cancer, who received NET and underwent surgery between June 2010 and December 2021, were recruited in this retrospective study. Eligibility criteria were patients who are ER-positive (nuclear staining ≥ 1% of cancer cells) and HER2-negative (immunohistochemical staining 0 or 1+, and negative fluorescence in situ hybridization for immunohistochemical staining 2+). Patients without pretreatment TILs data (n = 5), post-treatment Ki67 data (n = 1), or NET < 3 weeks (n = 10) were excluded. The ET agents included aromatase inhibitors (n = 123), ovarian function suppression plus TAM (n = 33), TAM (n = 11), and fulvestrant (n = 3). The median duration of NET was 5.2 months (range, 3 weeks to 92.8 months).

### Cut-off of Ki67 expression levels and definition of response to NET

We evaluated the average expression of Ki67 in the nuclei of cancer cells using immunohistochemistry with measurements by eyeballing. Pre-treatment Ki67 was divided into high (≥ 20%; n = 59) and low (< 20%; n = 111) groups. The response to NET was defined by post-treatment Ki67 using 2.7% as the cut-off value, following a previous study [5], and patients were classified as responders (≤ 2.7%; n = 81) and non-responders (> 2.7%; n = 89).

### Evaluation of TIL levels

TIL levels were determined in hematoxylin and eosin-stained samples obtained by core needle biopsy before NET and in surgically resected tissues after NET, as described in a previous study [16]. First, we microscopically identified lesions containing a relatively high number of invasive cancer cells and lymphocyte infiltration using a low-power field (×40). The hotspot with the highest lymphocyte infiltration was selected in a medium-power field (×100). Excluding neutrophils, eosinophils, and macrophages, lymphocytes and plasma cells in both the peritumoral and intratumoral stromal regions were evaluated. Finally, TIL levels were calculated as the percentage of areas involved in lymphocytes and plasma cells in the entire tumor and adjacent stroma. TIL levels were independently examined by two investigators (R. F. and T. W.), and in cases of discrepancies, they were discussed until a match was reached. Representative cases with low (1%) and high (30%) TIL levels are shown in Supplementary Fig. S1a and b, respectively.

### Immunohistochemical staining of CD8 and FOXP3 in TILs

Paired tumor samples before and after NET were available for 42 breast cancer cases. Using these samples, we immunohistochemically analyzed the expression of CD8 and FOXP3. Cell Conditioning Solution (Ventana Medical Systems, Inc., Basel, Switzerland) for 64 min and BOND Epitope Retrieval Solution 2 (Leica Microsystems, Tokyo, Japan) for 20 min were used for CD8 and FOXP3 antigen retrieval, respectively. Primary antibodies against CD8 (no dilution; CONFIRM anti-CD8 SP57 rabbit monoclonal antibody, Roche Diagnostics K.K., Tokyo, Japan) and FOXP3 (dilution 1:500; 236A/E7 antibody ab20034; mouse monoclonal; Abcam, Cambridge, UK) were used. Cell membrane and nuclear staining of lymphocytes were considered positive for CD8 and FOXP3, respectively (Supplementary Fig. S1c, d). Positive cells were counted at × 400 magnification, and the average counts of four fields were used to determine the cell counts in each sample, as previously reported [17].

### Measurement of absolute neutrophil and lymphocyte counts in peripheral blood during treatment

Neutrophil and lymphocyte counts in peripheral blood were measured automatically using a Sysmex hematology analyzer XN-9000 (Sysmex Corporation, Kobe, Japan). Data from paired samples obtained before and after NET were available for 151 patients. All blood samples were collected and measured within one month before the start of treatment or surgery. Neutrophil counts were calculated as the sum of the stab and segment fractions.

### Statistical analyses

The relationship between clinicopathological factors and the response to ET was analyzed using Fisher’s exact test or the Wilcoxon rank-sum test. TIL levels before and after NET and their changes were compared between responders and non-responders using the Wilcoxon rank-sum and Wilcoxon signed-rank tests, respectively. Relationships between TILs or response and CD8 + cells, FOXP3 + cells, or the FOXP3 + /CD8 + cell ratio were analyzed using the Wilcoxon rank-sum test or Wilcoxon signed-rank test. Changes in CD8 + cells, FOXP3 + cells, absolute neutrophil counts (ANC), and absolute lymphocyte counts (ALC) before and after NET were calculated using Wilcoxon signed-rank test. The OR and 95% CI for univariable and multivariable analyses were obtained using logistic regression models. All statistical analyses were performed using a two-sided test with JMP^®^ Pro Version 15 (SAS Institute Inc., Cary, NC, USA), and statistical significance was set at *p* < 0.05.

## Results

### Clinicopathological factors between responders and non-responders to NET

Table 1 shows the background of the patients according to their responses to NET. The number of responders was significantly higher in the Ki67-low group than in the Ki67-high group in pre-treatment samples (*p* = 0.001). Responses were more frequently observed in patients treated with aromatase inhibitors than in those treated with other endocrine agents (*p* = 0.039). There was no significant association between response and other factors, except for post-treatment progesterone receptor (PgR) levels (median, range: 1%, 0–100% for responders vs. 10%, 0–100% for non-responders; *p* = 0.012).

**Table 1:** The background of the patients according to their response (responders and non-responders) to neoadjuvant endocrine therapy

	Responder^b^	Non-responder^b^	*p* value
Menopausal status			
Premenopausal	16 (19.8%)	26 (29.2%)	0.160
Postmenopausal	65 (80.2%)	63 (70.8%)	
Primary tumor size			
T1	43 (53.1%)	37 (41.6%)	0.166
T2 and over	38 (46.9%)	52 (58.4%)	
Lymph Node Metastasis			
Negative	55 (67.9%)	66 (74.2%)	0.400
Positive	26 (32.1%)	23 (25.8%)	
Nuclear grade			
1	69 (85.2%)	73 (82.0%)	0.680
2 + 3	12 (14.8%)	16 (18.0%)	
Histological classification			
Invasive ductal carcinoma	78 (96.3%)	87 (97.8%)	0.670
Invasive lobular carcinoma	3 (3.7%)	2 (2.2%)	
ER % (Pre-treatment)^a^	95 (60–100)	95 (20–100)	0.100
ER % (Post-treatment)^a^	100 (50–100)	95 (20–100)	0.259
PgR % (Pre-treatment)^a^	60 (0–100)	70 (0–100)	0.857
PgR % (Post-treatment)^a^	1 (0–100)	10 (0–100)	0.012
Ki-67 (Pre-treatment)			
< 20%	63 (77.8%)	48 (53.9%)	0.001
≥ 20%	18 (22.2%)	41 (46.1%)	
Ki-67 (Post-treatment)			
< 20%	81 (100%)	67 (75.3%)	< 0.001
≥ 20%	0 (0%)	22 (24.7%)	
Endocrine therapy			
AI	65 (80.2%)	58 (65.2%)	0.039
Others	16 (19.8%)	31 (34.8%)	

^a^Median (Range)

^b^Responder: post-treatment Ki67 ≤ 2.7%, Non-responder: post-treatment Ki67 > 2.7%

### Relationship between TILs and response to NET

**Fig. 1:**
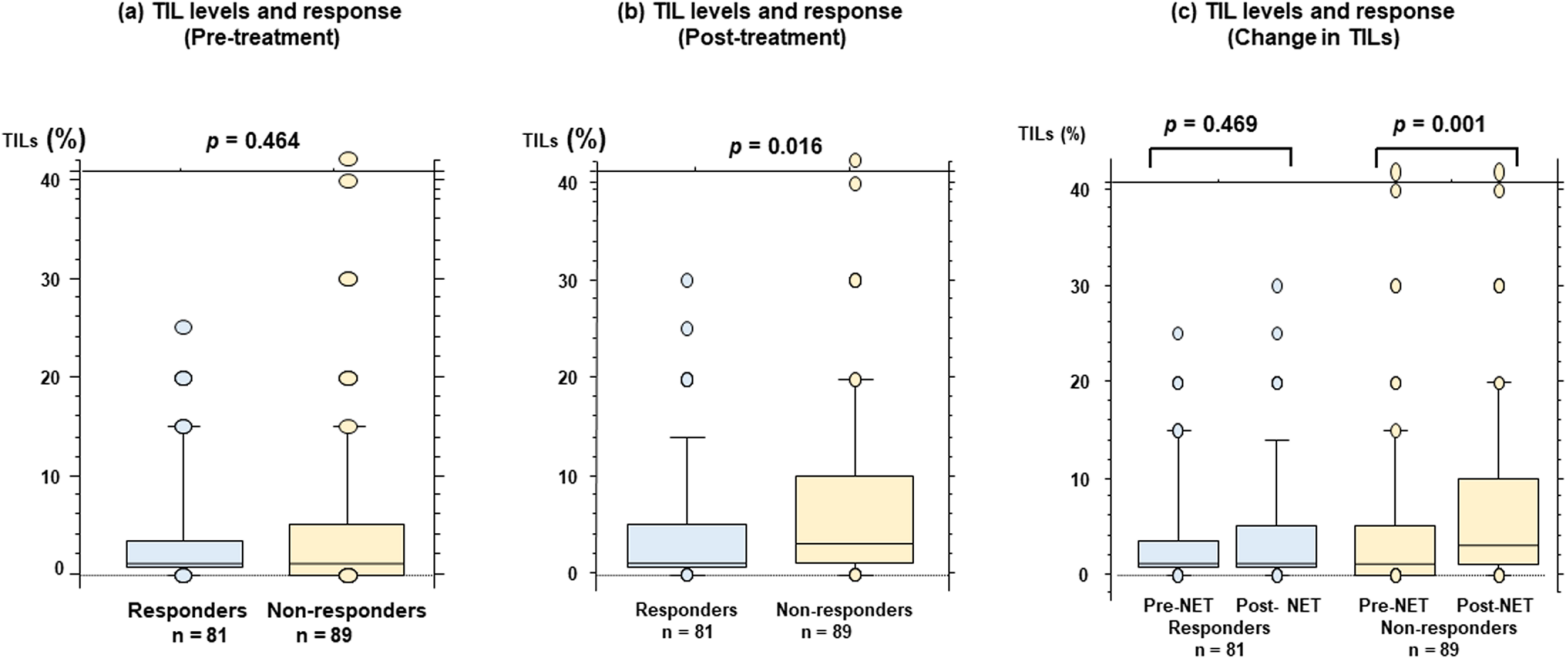
Association between tumor-infiltrating lymphocytes (TILs) and response to neoadjuvant endocrine therapy (NET). The levels of TILs are compared between responders and non-responders in pre-treatment samples (**a**) and post-treatment samples (**b**). Changes in TILs are compared according to response (**c**)

Pre-treatment TIL levels were not significantly different between responders (median, 1%; range, 0–25%; n = 81) and non-responders (median, 1%; range, 0–60%; n = 89; *p* = 0.464) (Fig. 1a). Meanwhile, the TILs of non-responders (median, 3%; range, 0–60%; n = 89) were significantly higher than those of responders (median, 1%; range, 0–30%; n = 81) in post-treatment tissues (*p* = 0.016) (Fig. 1b). A significant increase in TILs was observed in non-responders (*p* = 0.001), but not in responders (*p* = 0.469) (Fig. 1c).

### Univariable and multivariable analyses for response to NET

ER expression levels (OR 1.04; 95% CI 1.01−1.07 for 1% increase), Ki67 status (OR 0.33; 95% CI 0.17−0.65 for ≥ 20%), endocrine agents (OR 0.46; 95% CI 0.23−0.93 for non-AI), post-treatment TILs (OR 0.95; 95% CI 0.91−0.99 for 1% increase), and change in TILs (OR 0.48; 95% CI 0.26−0.90 for increased TILs) were significant predictors of response to NET by univariable analysis (Table 2). By multivariable analysis, TIL levels at post-treatment (OR 0.96; 95% CI 0.91−1.00; *p* = 0.055) were not significantly associated with response when adjusted for other factors, except for change in TILs. Meanwhile, TILs change (OR 0.44; 95% CI 0.21−0.89; *p* = 0.023), pre-treatment ER (OR 1.04; 95% CI 1.01−1.08; *p* = 0.009), and pre-treatment Ki67 (OR 0.28; 95% CI 0.12−0.61; *p* = 0.001) were significant and independent predictors of response to NET after adjusting for other factors, except for post-treatment TILs.

**Table 2:** Univariable and multivariable analyses for response to neoadjuvant endocrine therapy

	n (%)	Univariable analysis	*p* value	Multivariable analysis^b^	*p* value	Multivariable analysis^c^	*p* value
		Odds ratio (95%CI)^a^		Odds ratio (95%CI)^a^		Odds ratio (95%CI)^a^	
Menopausal status							
Premenopausal	42 (24.7%)	1	0.155	1	0.764	1	0.828
Postmenopausal	128 (75.3%)	1.68 (0.82–3.42)		0.83 (0.26–2.71)		0.88 (0.26–2.90)	
Tumor size							
T1	80 (47.1%)	1		1	0.075	1	0.169
T2 and over	90 (52.9%)	0.63 (0.34–1.15)	0.134	0.53 (0.27–1.07)		0.61 (0.31–1.23)	
Lymph node metastasis							
Negative	121 (71.2%)	1	0.369	1	0.168	1	0.135
Positive	49 (28.8%)	1.36 (0.70–2.64)		1.71 (0.80–3.65)		1.79 (0.83–3.85)	
Nuclear grade							
1	142 (83.5%)	1	0.579	1	0.400	1	0.304
2 + 3	28 (16.5%)	0.79 (0.35–1.80)		1.53 (0.57–4.12)		1.69 (0.62–4.56)	
ER % (Pre-treatment)		1.04 (1.01–1.07)	0.009	1.04 (1.01–1.07)	0.023	1.04 (1.01–1.08)	0.009
PgR % (Pre-treatment)		1.00 (0.99–1.01)	0.940	1.00 (0.99–1.01)	0.960	1.00 (0.99–1.01)	0.925
Ki-67 (Pre-treatment)							
< 20%	111 (65.3%)	1	0.001	1	0.003	1	0.001
≥ 20%	59 (34.7%)	0.33 (0.17–0.65)		0.30 (0.13–0.65)		0.28 (0.12–0.61)	
TILs (pre-treatment)		0.98 (0.95–1.02)	0.353				
TILs (post-treatment)		0.95 (0.91–0.99)	0.014	0.96 (0.91–1.00)	0.055		
TILs change							
Non-increase	102 (60.0%)	1	0.021			1	0.023
Increase	68 (40.0%)	0.48 (0.26–0.90)				0.44 (0.21–0.89)	
Endocrine therapy							
Aromatase inhibitor	123 (72.4%)	1	0.030	1	0.281	1	0.225
Others	47 (27.6%)	0.46 (0.23–0.93)		0.52 (0.16–1.72)		0.47 (0.14–1.58)	

^a^CI: confidence interval

^b^TILs change was excluded

^c^TILs of post-treatment was excluded

### Determination of CD8+ T-cell counts, FOXP3+ T-cell counts, and FOXP3+/CD8+ T-cell ratio according to response to NET or changes in TILs 

**Fig. 2:**
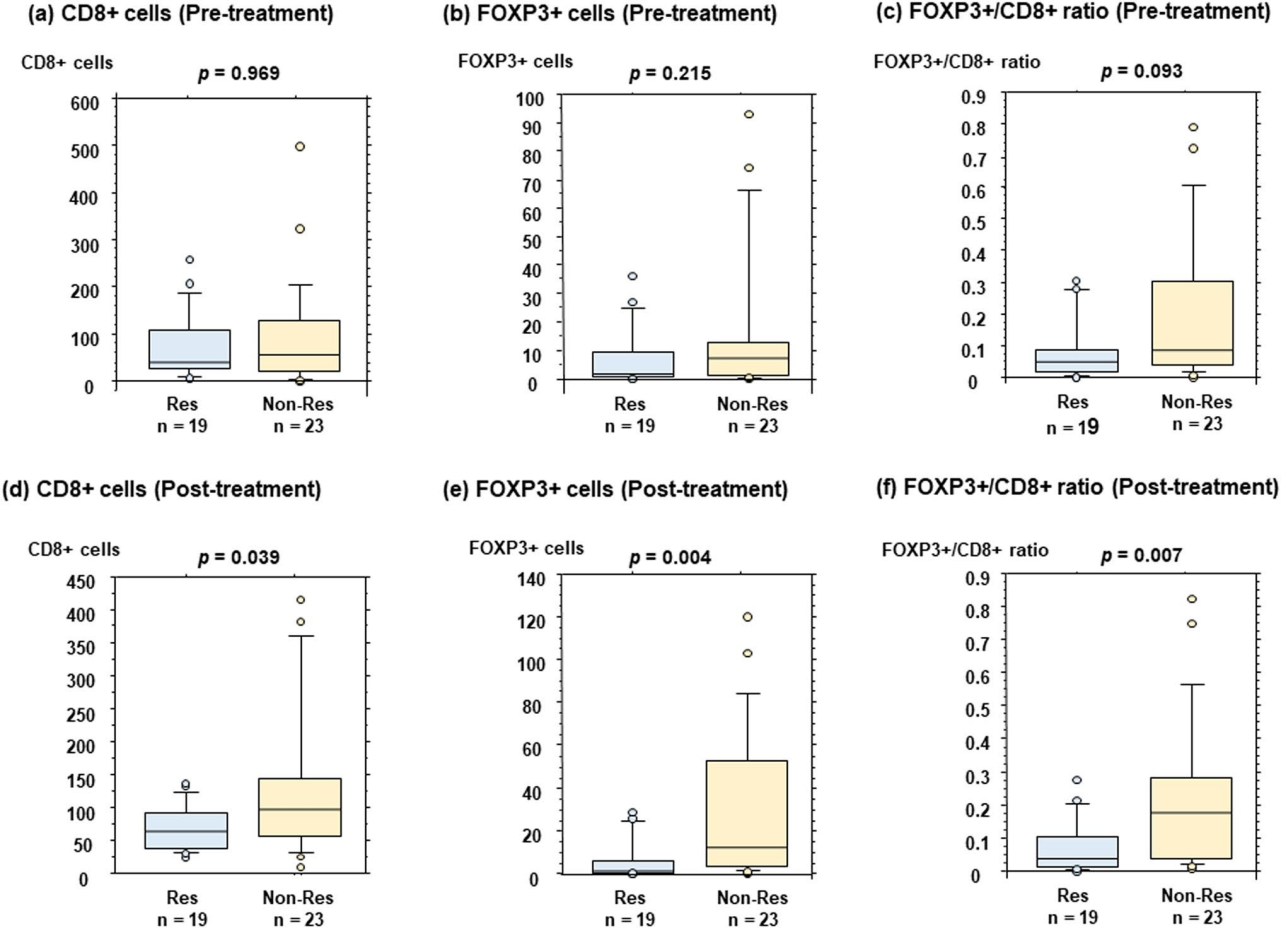
CD8 + and FOXP3 + T cell counts and FOXP3/CD8 T cell ratio are analyzed between responders (Res) and non-responders (Non-Res) to neoadjuvant endocrine therapy. In pre-treatment samples, the response and CD8 (**a**), FOXP3 (**b**), or FOXP3/CD8 ratio (**c**) are compared. Regarding post-treatment, the data for the CD8 (**d**), FOXP3 (**e**), and FOXP3/CD8 ratios (**f**) are shown

There was no significant association between the response to NET and CD8 + T cell counts (*p* = 0.969), FOXP3 + T cell counts (*p* = 0.215), or the FOXP3+/CD8 + T cell ratio (*p* = 0.093) in pre-treatment breast cancers (Fig. 2a–c). In contrast, CD8 + T-cell counts (*p* = 0.039), FOXP3+ T-cell counts (*p* = 0.004), and FOXP3+/CD8+  T-cell ratio (*p* = 0.007) were significantly higher in non-responders than in responders as for post-treatment breast cancers (Fig. 2d–f). Although the expression levels of CD8 + T cells and FOXP3 +  T cells were not significantly different before and after NET in responders (*p* = 0.586 for CD8 and *p* = 0.403 for FOXP3), CD8 + T cells and FOXP3 + T cells increased significantly after treatment in non-responders (*p* = 0.019 for CD8 and *p* = 0.005 for FOXP3) (Supplementary Fig. S2).

**Fig. 3:**
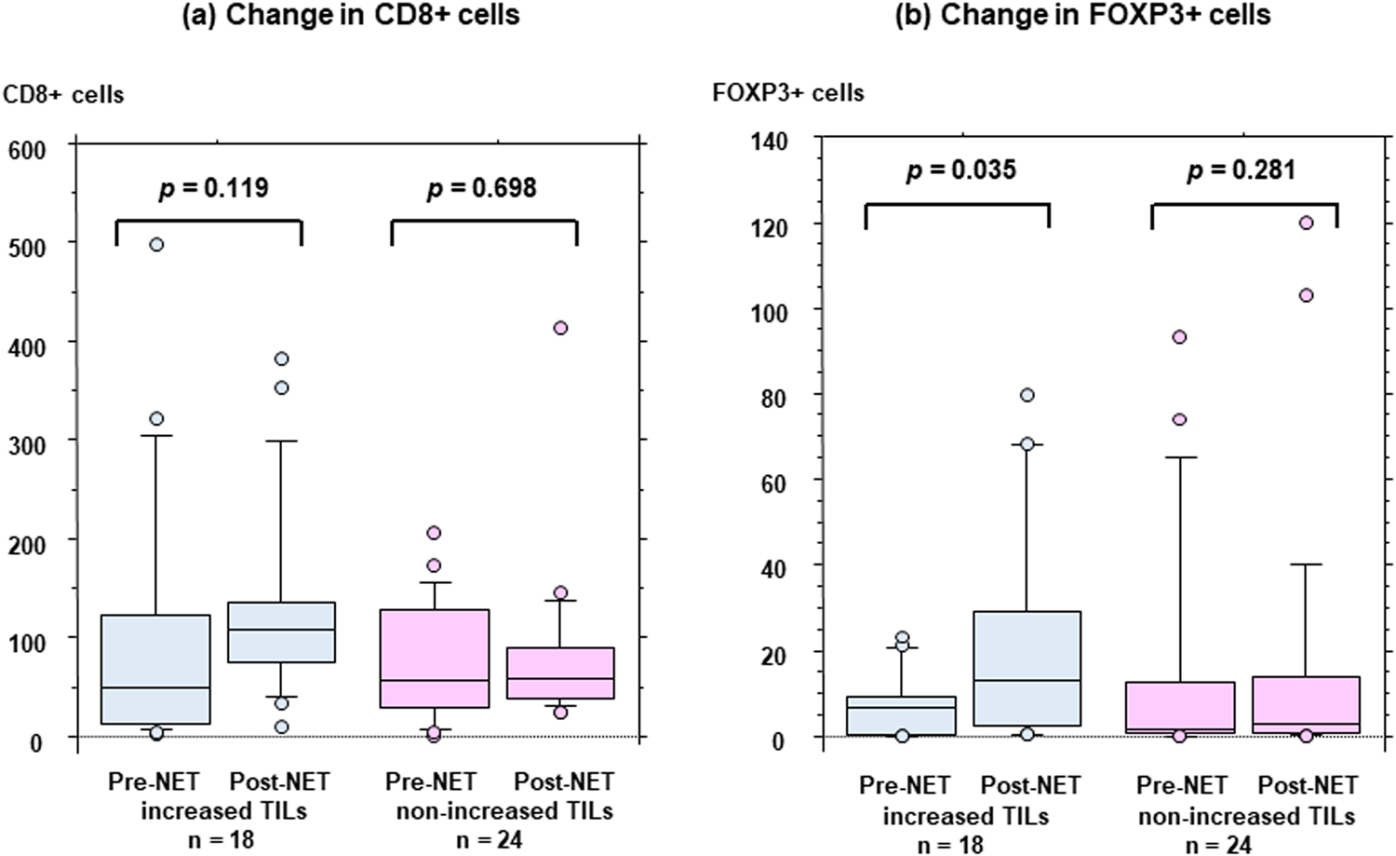
Changes in CD8 + (**a**) and FOXP3 + (**b**) T-cell counts between pre- and post-treatment with neoadjuvant endocrine therapy (NET) are evaluated according to breast cancer with increased and non-increased levels of tumor-infiltrating lymphocytes (TILs)

To clarify the mechanisms underlying the relationship between increased TILs and a poor response to NET, changes in CD8 + and FOXP3 + T cells during treatment were analyzed. Among patients with (n = 18) and without (n = 24) increased TILs groups, CD8 + T cells did not change after NET (median, range: 48.9, 3.0−498.5 vs. 107.3, 10.5 − 382.8, *p* = 0.119 and 56.5, 1–207.3 vs. 57.3, 24.5–415.0, *p* = 0.698, respectively) (Fig. 3a). In contrast, FOXP3 + T cells increased significantly after treatment in the increased TILs group (median, range: 7.0, 0−23.3 vs. 13.0, 0.5−79.8, *p* = 0.035) but not in the non-increased TILs group (median, range: 1.9, 0–93.3 vs. 3.3, 0–120.0, *p* = 0.281) (Fig. 3b).

### Changes in ANC and ALC to changes in TILs during NET

**Fig. 4:**
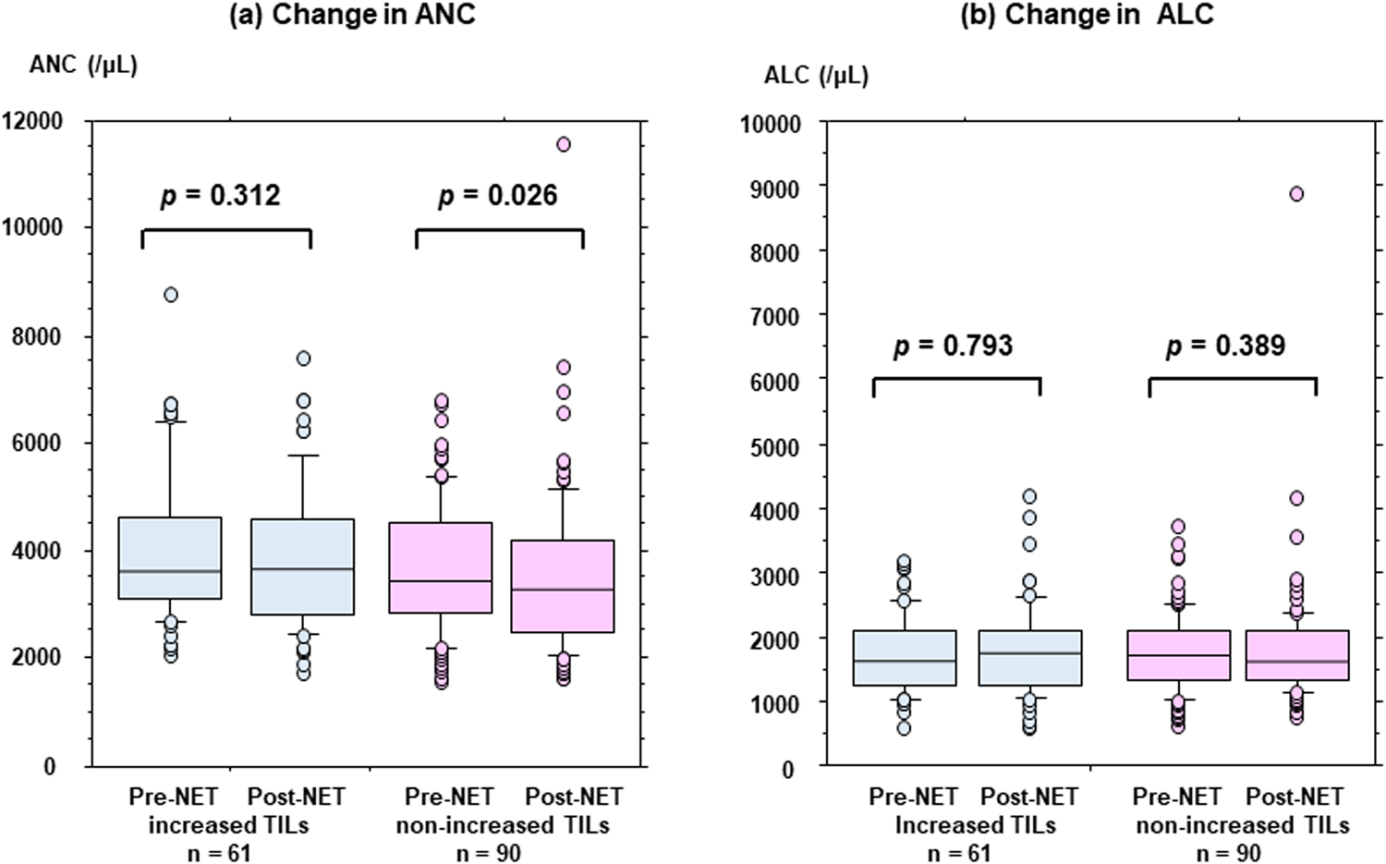
Changes in absolute neutrophil counts (ANC) (**a**) and absolute lymphocyte counts (ALC) (**b**) between pre-treatment and post-treatment with neoadjuvant endocrine therapy (NET) are compared according to breast cancer with increased and non-increased levels of tumor-infiltrating lymphocytes (TILs)

Changes in ANC and ALC levels before and after NET were analyzed considering TILs changes (Fig. 4). Among patients without increased TILs after treatment, ANC decreased significantly compared to that before treatment (median, range: 3417.8, 1568–6809 for pre-treatment and 3286, 1630–11,603 for post-treatment; *p* = 0.026) (Fig. 4a). In contrast, ANC did not change during NET in patients with increased TIL levels (median, range: 3635, 2038–8799 for pre-treatment and 3645, 1719–7602 for post-treatment; *p* = 0.312). There were no significant differences in ALC before and after NET in patients with (*p* = 0.793) or without (*p* = 0.389) increased TIL levels (Fig. 4b).

### Association between changes in TILs and clinicopathological factors

Breast cancer patients with increased TIL levels had larger tumor sizes (T2, 63.2% vs. 46.1%; *p* = 0.030) and more lymph node metastases positive (n-positive, 38.2% vs. 22.6%; *p* = 0.038) (Table 3). Regarding other factors, including menopausal status, ER and PgR expression levels, Ki67 status, and endocrine agents, no significant association was found with increased TIL levels. Furthermore, ANC and ALC were not significantly associated with the changes in TIL levels.

**Table 3:** The clinicopathological characteristics of breast cancer with and without increased TILs

	Increased TILs	Non-increased TILs	*p* value
Menopausal status			
Premenopausal	16 (23.5%)	26 (25.5%)	0.857
Postmenopausal	52 (76.5%)	76 (74.5%)	
Primary tumor size			
T1	25 (36.8%)	55 (53.9%)	0.030
T2 and over	43 (63.2%)	47 (46.1%)	
Lymph node metastasis			
Negative	42 (61.8%)	79 (77.5%)	0.038
Positive	26 (38.2%)	23 (22.6%)	
Nuclear grade			
1	54 (79.4%)	88 (86.3%)	0.292
2 + 3	14 (20.6%)	14 (13.7%)	
ER % (pre-treatment)^a^	95 (50–100)	95 (20–100)	0.606
ER % (post-treatment)^a^	100 (50–100)	95 (20–100)	0.869
PgR % (pre-treatment)^a^	70 (0–100)	60 (0–100)	0.808
PgR % (post-treatment)^a^	5 (0–100)	3 (0–100)	0.307
Ki-67 (pre-treatment)			
< 20%	40 (58.8%)	71 (69.6%)	0.188
≥ 20%	28 (41.2%)	31 (30.4%)	
Ki-67 (post-treatment)			
< 20%	57 (83.8%)	91 (89.2%)	0.354
≥ 20%	11 (16.2%)	11 (10.8%)	
TILs % (pre-treatment)^a^	1 (0–15)	1.5 (0–60)	0.0004
TILs % (post-treatment)^a^	5 (0–60)	1 (0–25)	< 0.0001
Neutrocytes/μL (pre-treatment)^a^	3635 (2038–8799)	3417.5 (1568–6809)	0.137
Neutrocytes/μL (post-treatment)^a^	3645 (1719–7602)	3286 (1630–11,603)	0.076
Lymphocytes/μL (pre-treatment)^a^	1670 (850–3168)	1660.5 (731–3697)	0.641
Lympnocytes /μL (post-treatment)^a^	1707 (600–4183)	1624 (741–8890)	0.869
Endocrine therapy			
AI	48 (70.6%)	75 (73.5%)	0.728
Others	20 (29.4%)	27 (26.5%)	

^a^Median (Range)

## Discussion

In this study, we found that increased levels of TILs after NET were significantly associated with a poor proliferative response in ER+/HER2- breast cancer (*p* = 0.001). Multivariable analysis showed that increased TIL levels after NET was an independent and significant predictor of poor treatment efficacy. Among patients with increased TILs, FOXP3 + T cells were significantly upregulated (*p* = 0.035) but not in patients without increased TILs (*p* = 0.281). Furthermore, a significant decrease in ANC in the peripheral blood was prominent among patients without increased TILs (*p* = 0.026) but not among those with increased TILs (*p* = 0.312). Based on these data, a mechanism by which the immunosuppressive milieu in breast cancer with increased TILs after ET results in inferior efficacy was speculated. As described in the Introduction, the study by Skriver et al. is consistent with our observation (TILs increased in patients with breast cancers with no pathological response) [14], whereas TILs increased in responders as demonstrated in Liang et al.’s report [15]. Although the detailed reasons for the discrepancy in the results are unknown, different methods of response evaluation, that is, the response was pathologically and radiologically evaluated in the studies by Skriver et al. and Liang et al., respectively, or different agents of ET, that is, letrozole in the former study and anastrozole or fulvestrant in the latter study, may be involved.

In a study of 987 ER+/HER2− breast cancers, higher TIL levels were significantly associated with lymph node metastases (*p* = 0.003), high tumor grade (*p* < 0.0001), low ER levels (*p* < 0.0001), and high Ki67 levels (*p* < 0.0001) [18]. The possibility that these aggressive phenotypes of breast cancer with high TIL levels are linked to a lower sensitivity to ET cannot be ruled out. However, in our study, NET response was not associated with lymph node metastasis, nuclear grade, and ER levels, except Ki67 levels (Table 1). Since a change in TIL levels was an independent predictor of response to NET by multivariable analysis, including Ki67 (Table 2), TIL level changes might not be associated with sensitivity mediated through clinicopathological factors of TIL-high tumors. The different roles of TILs in ER + /HER2− compared with those in HER2 + or TN breast cancers seem to stem from the composition of subsets of T cells, including FOXP3 + T cells, which are more abundant in ER + than in ER- breast cancers [19]. The immune suppressive function of FOXP3 + T cells in ER + breast cancers was further shown in the meta-analysis study, in which high tumor-infiltrating FOXP3 + T cells had shorter OS in the ER + (HR 0.86; 95% CI 0.77 − 0.96; *p* = 0.009), but not in the ER− (HR, 1.09; 95% CI 0.82−1.45; *p* = 0.569) breast cancers [20]. Therefore, the immunosuppressive function directed through FOXP3 + T cells may play an essential role in the biology of ER + breast cancer.

In a previous NET study, the CD8+/Treg T cell ratio was significantly increased in responders (*p* = 0.001) but not in non-responders (*p* = 0.744) [21]. Although both CD8 + and FOXP3 + T cells increased significantly after treatment in non-responders (Supplementary Fig. S2), FOXP3 + T-cell counts and the FOXP3+/CD8 + T cell ratio appeared to be superior to the CD8 + T cell counts for predictive efficacy. Because increased TILs in non-responders were accompanied by upregulated FOXP3 + T cells, we speculate that not CD8+, but FOXP3 + T cells plays an essential role in the efficacy of ET. Estrogen directly stimulates FOXP3 expression and function of Tregs in cervical cancer [22]. Generli et al. have reported a significant reduction in the number of Tregs after letrozole treatment [23]. Since this reduction in Tregs was restricted to the responder group, the response to letrozole was speculated to be mediated by Treg suppression. Similarly, among patients with ER+/HER2− metastatic breast cancer treated with CDK4/6 inhibitors and endocrine agents, a greater reduction in Tregs has been reported in responders than in non-responders [24]. These data support the idea that reduction in the number of Tregs induced by endocrine-based therapy plays an essential role in achieving a response. A meta-analysis documented the relationship between high NLR and poor prognosis in early breast cancer [25]. Increased myeloid-derived suppressor cells in peripheral blood have been reported to be significantly associated with a high NLR [26]. Furthermore, higher NLR significantly upregulates inflammatory cytokines, including IL-6 and IL-8, in colorectal cancer [27]. These data strongly support the hypothesis that the altered ratio of neutrophils to lymphocytes reflects an immunosuppressive microenvironment in the tumor.

The detailed mechanism by which the number of FOXP3 + T cells increases in non-responders after ET remains unknown. Tregs are induced by several factors, including hypoxia and transforming growth factor-β (TGF-β) signaling [28, 29]. Overexpression of the TGF-β metagene has been reported in immune-rich ER + breast cancers [30] and upregulation of TGF-β by treatment with an aromatase inhibitor [31]. As TGF-β signaling has been reported to be related to resistance to letrozole or TAM [32, 33], upregulation of this signaling mediated through the resistance process to ET might activate Tregs functions. The observation that increased TILs after NET frequently occur in breast cancers with large tumor sizes and lymph node metastases (Table 3) may indicate an unfavorable immune microenvironment for these tumors.

The results obtained here are expected to contribute not only to the understanding of the mechanisms of poor response to ET induced by the microenvironment of high TILs but also to the identification of patients with inferior efficacy to ET. If sensitivity to ET is predicted more precisely by a combination of TILs change and Ki67 suppression, this prediction model will be clinically useful and will lead to the development of a new treatment strategy that combines molecules that modulate the immune microenvironment, including immunotherapy.

The present study had several limitations. Since the evaluation of TILs in whole tumors from pre-treatment is not feasible, TILs counts were evaluated not by average but by a hotspot, following the previously published method by Hida et al. [34]. Depending on the method used, the increased TIL levels were possibly caused by a selection bias of the hotspot lesions. However, TILs increases were observed in non-responders but not in responders, which might deny this bias. In addition, determining post-treatment Ki67 ≤ 2.7% as a responder seems to be inappropriate for tumors with Ki67 ≤ 2.7% at baseline. Although eight such cases were included in the present study, we confirmed consistent results when these cases were excluded from the analyses (data not shown). We believe that the current methodological issues did not influence the results obtained. Since the number of patients was not enough, further studies with a larger sample size including analyses of factors related to Treg function, such as hypoxic conditions and activation of TGF-β signaling are needed.

## Conclusions

This study showed that an increase in TILs was significantly associated with poor proliferative response to NET in ER+/HER2− breast cancers. It is speculated that the upregulation of FOXP3 + T cells after NET results in inferior sensitivity. These data indicate an essential role for ET not only in tumor suppression but also in the immune response. The poor prognosis of patients with high TIL levels in ER+/HER2− breast cancer might be partially explained by poor sensitivity to ET.

### Supplementary Information

Below is the link to the electronic supplementary material.

Supplementary file1 (DOCX 1464 KB)

## Data Availability

Data from individual participants were unavailable because the ethics committee did not permit their publication.
