# Supplementary material for: An increase in tumor-infiltrating lymphocytes after treatment is significantly associated with a poor response to neoadjuvant endocrine therapy for estrogen receptor-positive/HER2-negative breast cancers
Source: Breast Cancer. 2023 Apr 28;30(5):703–13. doi: 10.1007/s12282-023-01462-5 (PMC10404203; doi:10.1007/s12282-023-01462-5)
Supplement: Supplementary file 1 — Supplementary file1 (DOCX 1464 KB) [file 12282_2023_1462_MOESM1_ESM.docx]

**
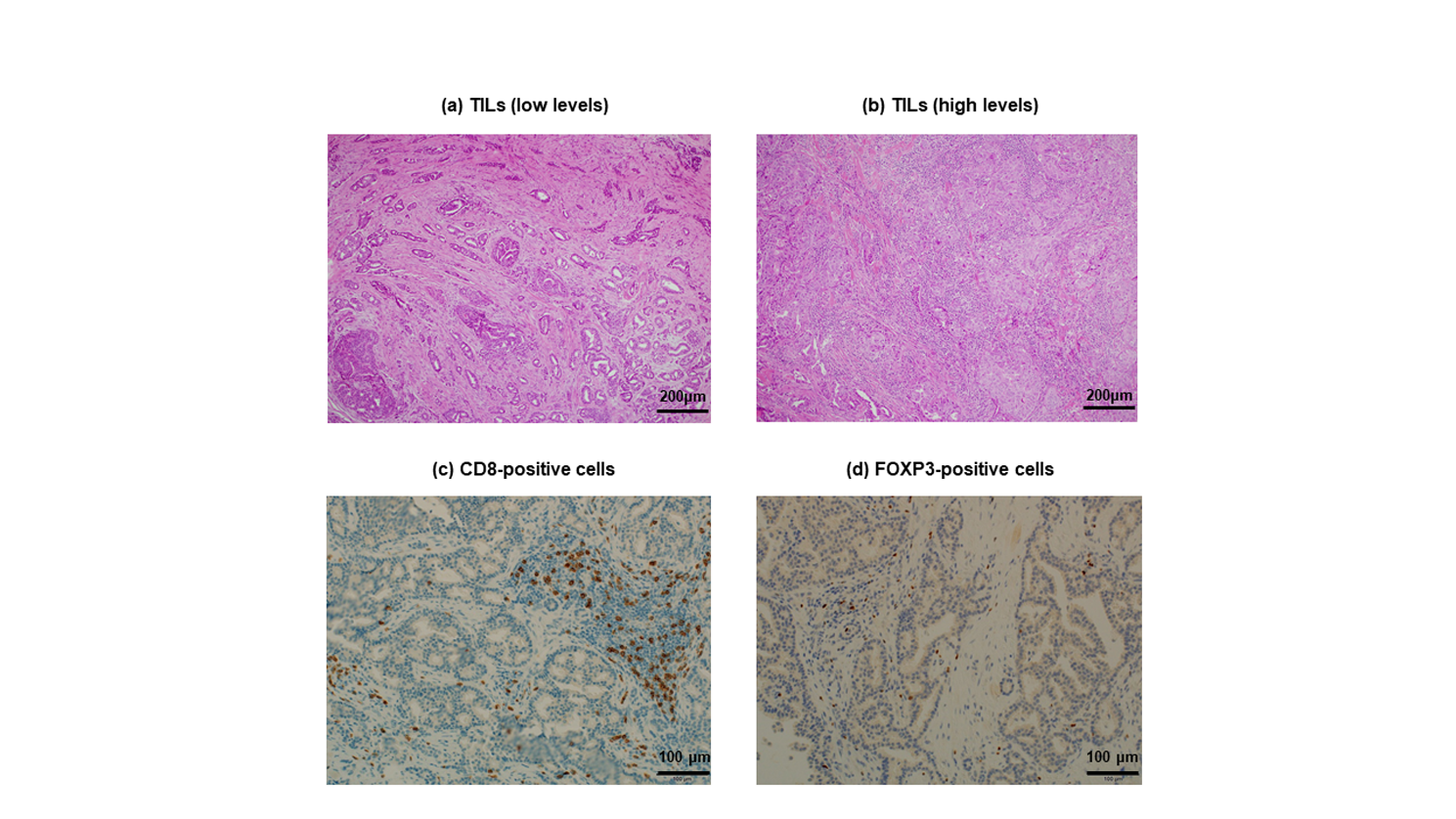
**

**Fig. S1**

Examples of tumor-infiltrating lymphocytes (TILs) and CD8 and FOXP3 immunohistochemical staining. Tumors with low levels of TILs (1%) (a) and high levels of TILs (30%) (b). Positive immunohistochemical staining of CD8 (c) and FOXP3(d). Membrane staining for CD8 and nuclear staining for FOXP3 are evaluated.

**
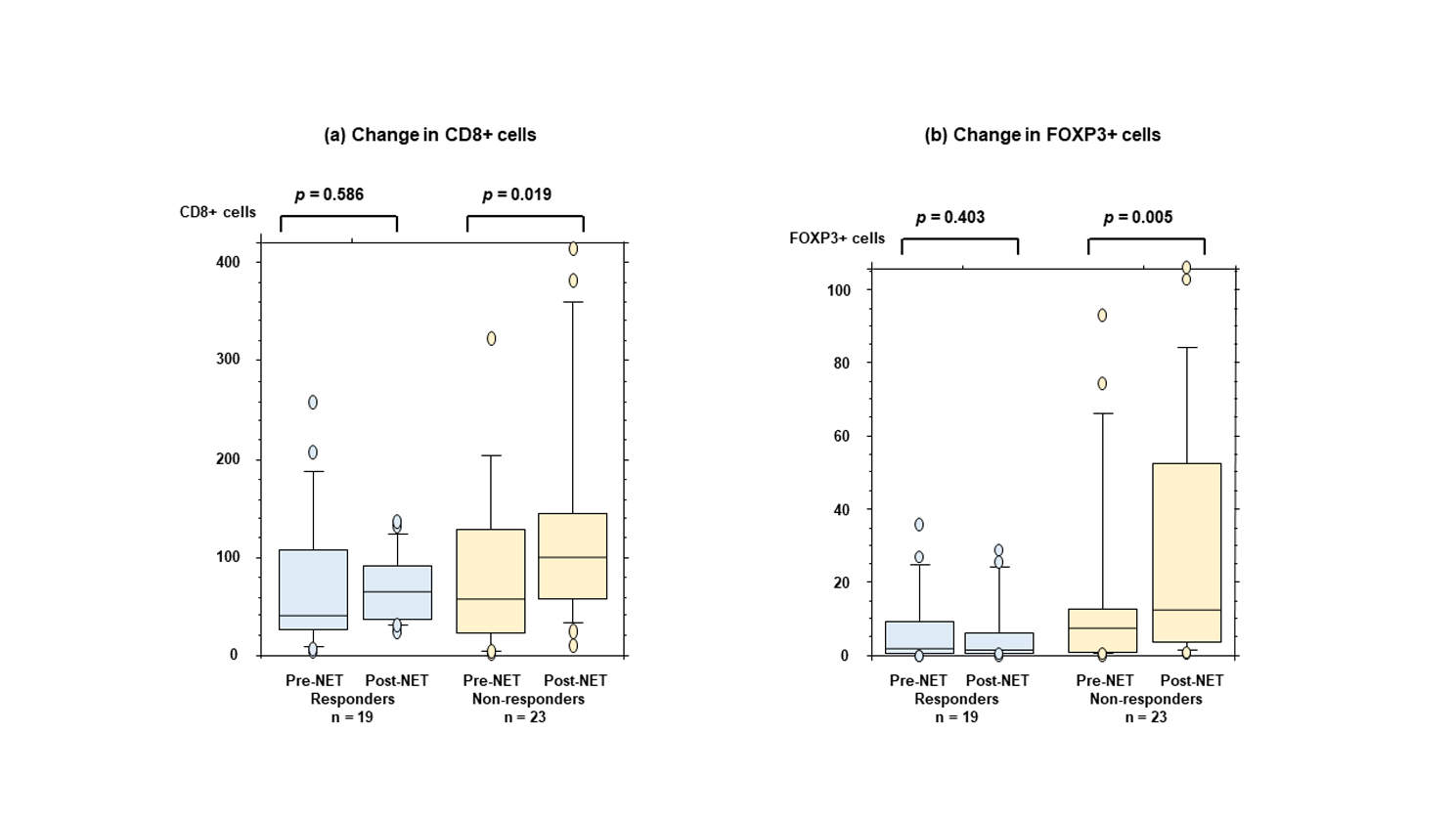
**

**Fig. S2**

Changes in CD8+ (a) and FOXP3+ (b) cell counts between pre-treatment and post-treatment with neoadjuvant endocrine therapy (NET) are evaluated according to the response.
